# Evaluation of Bending Deformations in Slender Cylindrical Structures Using Distributed Optical Fibre Strain Sensing

**DOI:** 10.3390/s25237366

**Published:** 2025-12-03

**Authors:** Madhubhashitha Herath, Oleg V. Ivanov, Kaushal Bhavsar, James M. Gilbert

**Affiliations:** 1 School of Engineering and Technology, Faculty of Science and Engineering, University of Hull, Hull HU6 7RX, UK; k.bhavsar@hull.ac.uk; 2 Energy and Environment Institute, Faculty of Science and Engineering, University of Hull, Hull HU6 7RX, UK

**Keywords:** distributed optical fibre sensing, bending deformation, strain measurement, slender cylindrical structures, Brillouin optical time-domain reflectometry, cable bending

## Abstract

Structures with slender cylindrical geometries, such as subsea power cables are critical components of infrastructure systems. These structures are prone to bending deformation under load, which can ultimately cause structural failure. In this study, distributed optical fibre sensors are used to monitor the bending deformation in slender cylindrical structures. Brillouin optical time-domain reflectometry-based strain sensing was used to experimentally study three-point bending and approximately constant curvature bending of a 6 m long circular hollow section (CHS). Optical fibres were attached to the outer surface of the CHS in two different configurations: parallel to the longitudinal axis and helically wound around the CHS. Strain responses due to changing magnitudes of deformation and changing orientation of the optical fibre around the circumference of the CHS were studied. A finite element model was employed to simulate and interpret the observed strain responses. A strain response inverse analysis was conducted using the strain data obtained from the experimental study to reconstruct the deformed shapes of the CHS. Both the longitudinally aligned and helically wound fibres showed distinct strain profiles that differentiate the three-point bending and constant curvature bending behaviours. The results revealed the ability of optical fibre sensing to evaluate the type; magnitude; and orientation of the bending deformations. This fundamental understanding supports the design of sensing systems for critical cylindrical infrastructure.

## 1. Introduction

Slender cylindrical structures, such as pipelines, power cables and communication cables, are vital infrastructure components with significant social and economic impacts. Optical fibre sensors are widely regarded as effective tools for monitoring the condition and remaining lifespan of these critical assets [1,2,3,4,5]. Compared to point sensors such as Fiber Bragg Grating (FBG) sensors, Distributed Optical Fibre Sensors (DOFS) are more effective for monitoring elongated structures, as they can measure over long distances using a standard optical fibre [6]. Distributed temperature (DTS) [7,8], acoustic (DAS) [9,10], and strain (DSS) [11,12] sensing technologies are commonly used for monitoring cylindrical infrastructure.

Cylindrical structural members subjected to bending exhibit varying strain behaviour due to their geometry. The axial strain on the outer surface varies along the length of the member and across its thickness, being tensile on the convex side and compressive on the concave side [13]. Understanding this radial and axial strain interaction is critical for accurate structural analysis. DAS, DSS, and hybrid systems are employed to assess the bending behaviour of cylindrical structures, providing complementary measurements of dynamic and static strain responses [14,15,16].

Optical fibres can be attached either parallel to the longitudinal axis or helically wound around cylindrical members [17]. Sasaki et al. conducted distributed strain sensing on a steel pipe-based well mockup subjected to three-point bending using Brillouin optical time-domain reflectometry/analysis (BOTDR/A) and optical frequency-domain reflectometry (OFDR) fibre optic interrogators. Their results indicate that the tight-buffered optical cable is the most suitable for strain sensing, exhibiting maximum strain measurement errors of −36% and −24% at the peak elastic and plastic bending loads, respectively, compared to conventional sensors. In contrast, the non-tight-buffered optical cable showed significantly higher errors of −45% and −71%, respectively [15].

Zhang et al. employed a Brillouin sensor in combination with the conjugate beam method to analyse the deformation of a 4 m long pipeline. BOTDA was used to obtain strain measurements, achieving a spatial resolution of 50 cm, with a sampling spacing of 10 cm [11]. In another study, multiple optical fibres attached parallel to the axis of a cylindrical tube were used to detect the tube’s bending resulting from ground displacement. Using optical backscattered reflectometry (OBR), a minimum detectable displacement of 0.2 mm was achieved [18].

Masoudi et al. demonstrated that a distributed optical fibre vibration sensor can effectively map dynamic strain along subsea power cables. The sensing system achieved a spatial resolution of 1 m and a strain resolution of 4 microstrains (με) over a 10 km sensing range. Experimental results indicated that the sensing fibre is subjected to less than 4% of the total bending strain experienced by the cable, primarily attributed to the helical configuration of the optical fibre within the cable, along with provisions that mechanically decouple the fibre from the cable structure [14]. A hybrid sensing approach was employed by Ryvers et al., combining FBGs bonded to the sheath of a fibre optic cable with a second fibre housed in a loose tube and interrogated using OFDR [16]. This configuration was used to analyse both constant-curvature and variable-curvature bending scenarios of a power cable.

Distributed optical fibre strain sensing for shape sensing and reconstruction has been studied across various applications, including biomedical, civil infrastructure, aerospace, and marine engineering [19,20]. Real-time shape sensing offers critical insights into the structural integrity and operational conditions of marine structures [21]. Advanced shape reconstruction models, combined with strain data obtained from axially or helically wound optical fibres, can be effectively utilized to accurately reconstruct the real-time shape of cylindrical structures [22,23]. Theories such as geometrically exact beam theory, a general-purpose framework for nonlinear analysis of slender structures [24], can be further explored for shape sensing in highly flexible engineering structures.

This research focuses on evaluating bending deformations in slender cylindrical structures using BOTDR-based distributed optical fibre sensing system, aiming to advance technology for subsea power cable monitoring. BOTDR enables accurate, distributed static and quasi-static strain sensing over long ranges, ideal for assessing true bending strain. BOTDR provides absolute strain measurements, critical for shape reconstruction, unlike DAS, which captures strain rate for dynamic events. BOTDR also distinguishes temperature and strain without auxiliary fibres. While OFDR suits short-range, high-resolution needs, BOTDR offers centimetre- to meter-level resolution over extended lengths (up to tens of km), making it optimal for the monitoring of slender cylindrical structures.

During the bending tests, strain along the sensing optical fibre was measured using a “VIAVI FTH-9000” Brillouin optical time-domain reflectometer (BOTDR). Brillouin scattering is a nonlinear effect involving the interaction between optical and acoustic fields in optical waveguides. It is useful in the field of photonics, where it supplies a tunable ultra-narrow linewidth response that can be used for engineering applications, including sensing. BOTDR technology is a distributed optical fibre sensing technology based on self-released Brillouin backscatter. When the pulse of light is incident from one end of the optical fibre at a certain frequency, the light propagating in the optical fibre generates scattered light at a shifted wavelength. The Brillouin scattering light returns to the incident end along the optical fibre. When the optical fibre is subjected to deformation or temperature variation, the Brillouin frequency shift (BFS) is observed, and the BFS is linearly related to the strain and temperature variation in the sensing optical fibre.

The Brillouin frequency shift can be expressed by Equation (1) when the strain and temperature of the fibre at a certain length are changing [25].

(1) $$\Delta \upsilon_{B}\left(x\right)=C_{1}\hat{A}\cdot \Delta \varepsilon \left(x\right)+C_{2}\cdot \Delta T(x)$$

In the equation, $\Delta \upsilon_{B}\left(x\right)$, $\Delta \varepsilon \left(x\right),$ and ΔT(x) are the BFS, strain variation, and temperature variation of the sensing fibre at x meters. $C_{1}$ and $C_{2}$ are the strain and temperature coefficients of the sensing optical fibre.

For every point along the sensing fibre, BOTDR acquires the Brillouin spectrum and determines the resonant Brillouin frequency shift (BFS). The temperature or strain variations are determined by considering the strain and temperature coefficient of the BFS. Depending on the application, VIAVI FTH-9000 BOTDR offers three modes to exploit the acquired data: temperature only, strain only, and both temperature and strain.

In this study, a CHS was examined under three-point bending. In addition, an approximately constant curvature was examined using a seven-point bending configuration. Two fibre optic configurations were employed: one with the fibres aligned parallel to the CHS’s axis and another with the fibres helically wound around the CHS. The strain in the fibres was measured using BOTDR for changing the magnitude, shape, and orientation of the bending deformations of the CHS. Finite element analysis was performed to compare the results obtained from the CHS bending experiments. Finally, a strain response inverse analysis was conducted using the strain data obtained from the experimental study to reconstruct the deformed shapes of the CHS.

This research lays the groundwork for monitoring slender cylindrical structures, such as subsea power cables, using BOTDR technology. This study compares two fibre layout configurations: parallel and helical. Understanding the effects of these differences is critical for power cable monitoring. It also evaluates the strain response of circular sections under both three-point bending and constant curvature bending, providing a direct comparison using the same CHS and bending test rig. Unlike most distributed strain sensing research that focuses on small deformations, this work captures large deformations in long, slender sections. Additionally, this paper explores the capabilities of BOTDR for absolute strain measurement in elongated structures, identifies limitations such as spatial averaging effects in BGS, and offers guidance on selecting appropriate optical pulse and helix pitches. This work also applies existing theoretical principles to differentiate phase shifts in strain profiles measured using helically wound fibres, introducing a novel method for deformation evaluation.

## 2. Experimental Setup and Procedure

The following section describes the materials, specimen fabrication, bend test procedure and distributed optical fibre sensing employed for the experimental study. A CHS was used to study the slender cylindrical structures subjected to bending deformations. The 6 m long CHS was made by joining two commercially available 3 m long polypropylene plastic pipes manufactured by Polypipe, UK (BS EN 1451-1:2017 [26]). A custom designed internal socket made of glass fibre-reinforced polymer composite was used to join the pipes. Hence, the joint’s outer surface was sufficiently smooth, ensuring no adverse interaction with the attached optical fibres. The measured inner and outer diameters of the CHS were 37.5 mm and 40 mm, respectively. According to the average material property data published by the MatWeb database for extrusion-grade polypropylene, the material exhibits a density of 0.918 g/cm^3^, an ultimate tensile strength of 66.4 MPa, an elongation at break of 272%, an elongation at yield of 12.8%, a modulus of elasticity of 1.40 GPa, a flexural yield strength of 36.0 MPa, and a flexural modulus of 1.39 GPa [27].

**Figure 1:**
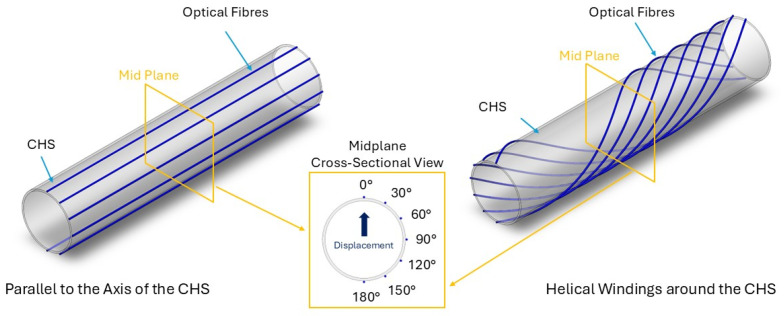
Configurations of the sensing optical fibre attached to the outer surface of the CHS: parallel to the axis of the CHS and in helical windings around the CHS.

As shown in Figure 1, sensing optical fibres were attached to the outer surface of the CHS in two configurations: parallel to the axis of the CHS and in helical windings around the CHS. A pitch of 2 m was applied to the helical windings, resulting in a helix angle of approximately 86.4°.

A single mode “CORNING-SMF28e-Ultra” (Corning Inc., USA) fibre was used as the distributed optical fibre sensor. The glass cladding and acrylate coating diameters of the optical fibre were 125 μm and 242 μm, respectively. A 100 m long jacketed fibre (THORLABS SMF 28, Thorlabs Inc., USA) was used to connect the sensing optical fibre to the BOTDR. The sensing fibre and the connecting fibre were spliced and connected to the BOTDR using a SC/APC connector.

Two types of adhesion methods were used to attach the optical fibre sensor to the surface of the CHS. In the first method, a double-sided adhesive tape manufactured by 3M, UK with a polyester carrier and a modified acrylic adhesive film liner was attached on the CHS along a predetermined path. Subsequently, the optical fibre sensor was attached to the double-sided adhesive tape. Afterwards, a PVC insulation tape coated with cross-linked solvent-based rubber resin adhesive was placed on top of the optical fibre sensor as a cover to protect the sensor. In the second method, an epoxy-constituent-based rapid adhesive (Araldite) produced by Huntsman Advanced Materials, UK was used to attach the optical fibre sensor. The optical fibre sensor was laid on the CHS along a predetermined path and it was covered with the adhesive, which was allowed to cure at room temperature for 3 h.

**Figure 2:**
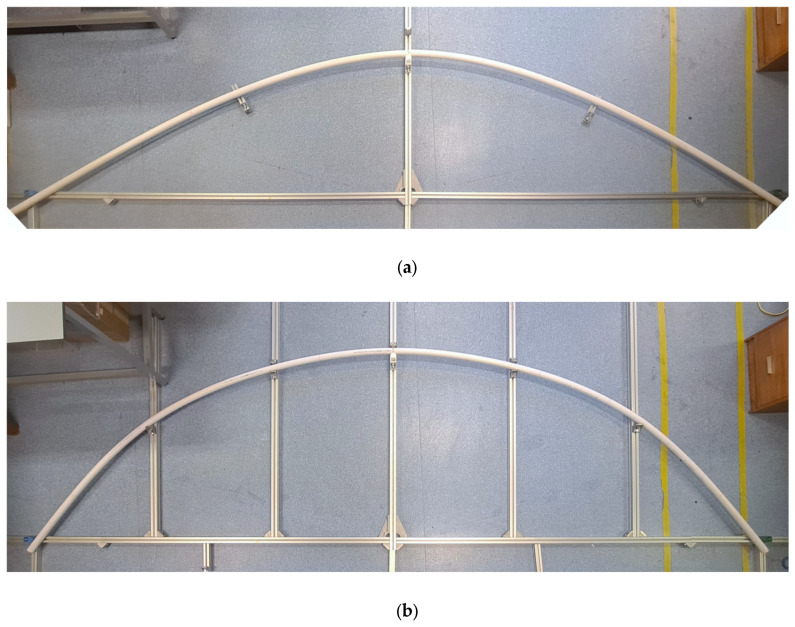
Bending configurations on the test rig: (**a**) three-point bending and (**b**) constant curvature bending.

A custom-made test rig was used to bend the CHS under three-point bending (3PB) and constant curvature bending (CCB) arrangements. The test rig was constructed using 40 × 40 mm sized aluminium profile struts assembled with aluminium angle brackets. The assembled test rig (6 × 2 × 0.04 m^3^) was placed horizontally on a laboratory floor. The bending configurations of the CHS on the test rig are shown in Figure 2. Custom-designed sliding supports were fabricated by 3D printing and used to force and fix the CHS on the test rig into the predetermined bend shapes.

**Figure 3:**
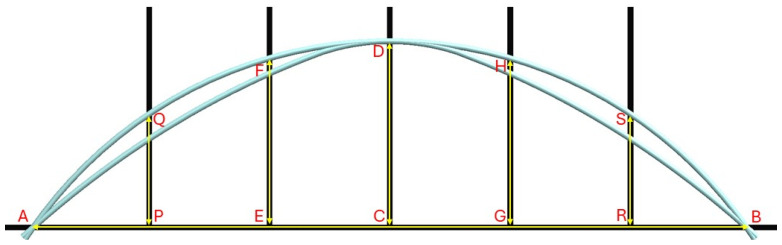
Schematic diagram of the test rig and the variable dimensions for different levels of bending deformations.

Figure 3 illustrates a schematic diagram of the test rig. The fixed and variable dimensions of the test rig are labelled in Figure 3. In the experimental setup, an approximately constant curvature was achieved using a seven-point bending configuration. While not perfectly uniform, the resulting curvature closely approximated ideal constant curvature bending. In Phase 1, both three-point bending and constant curvature bending tests were conducted under 5 levels of bending deformations. The fixed and variable dimensions on the test rig for the five different levels of bending deformations are presented in Table 1.

In Phase 2, a series of three-point bending tests were conducted to study the bending direction by changing the mid plane intersecting point of the optical fibre sensor. As illustrated in Figure 1, the top dead centre of the mid plane was considered as 0°, and rotational angles of 30°, 60°, 90°, 120°, 150°, and 180° were considered for the experiment. For these three-point bending tests, Level 1 bending deformation was employed.

During Phase 1, optical fibre sensors were attached using double-sided adhesive tape and demonstrated reliable measurement of tensile strains but exhibited limited sensitivity to compressive strains. This limitation is attributed to compromised strain transfer under compressive loading, likely caused by tape wrinkling or loss of adhesion between the tape and the CHS surface. To address this issue, Phase 2 experiments employed an epoxy-based adhesive to mount the optical fibres, particularly when testing at varying rotational angles. The use of epoxy significantly enhanced strain transfer efficiency, enabling accurate detection of both tensile and compressive strains.

In this study, the bending experiments were conducted at room temperature. The BOTDR system was operated in strain-only mode, employing a strain coefficient ${(C}_{1})$ of 0.048 MHz/με. The optical pulse and resolution of the scan were 5 ns and 0.5 m, respectively. A relatively high BFS was anticipated during the experiments due to the large strains caused by the large deformation. Therefore, the range of the Brillouin acquisition was increased by setting the start and end frequencies to 10.25 GHz and 11.35 GHz.

## 3. Strain Measurements

This section presents the results and corresponding data analysis of the experimental study. The analysis includes both three-point and constant curvature bending, based on the measured data from fibre mounted parallel to the axis and wound helically around the CHS. The orientation of the bending deformations was also analysed to provide insights into directional behaviour. For easier data interpretation, the midpoint of the CHS along its length was designated as 0 m. The two ends of the CHS are represented as −3 m and +3 m, respectively.

**Figure 4:**
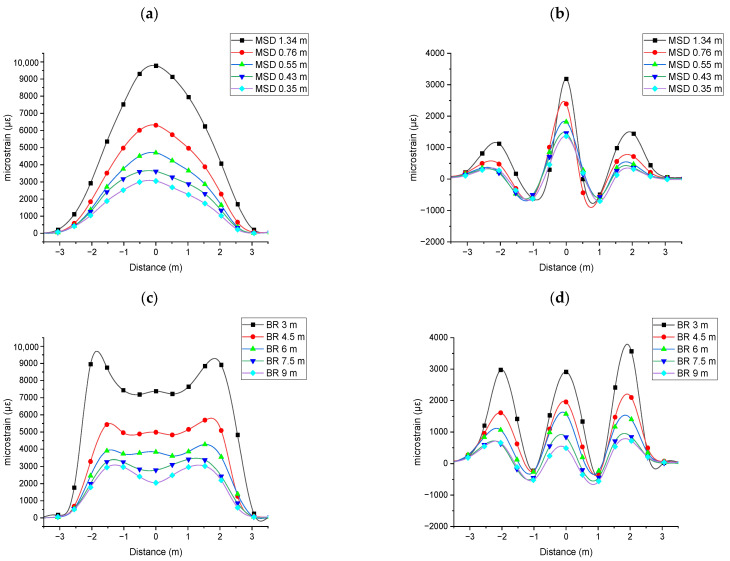
Experimental strain data of the CHS subjected to bending deformations: (**a**) three-point bending with fibre parallel to the axis of the CHS; (**b**) three-point bending with fibre in helical winding around the CHS; (**c**) constant curvature bending with fibre parallel to the axis of the CHS; (**d**) constant curvature bending with fibre in helical winding around the CHS.

Both the three-point bending and constant curvature bending tests were conducted at five levels of deformation. For ease of reference, deformation levels in the three-point bending test are represented by midspan displacement (MSD), while in the constant curvature bending test, they are represented by bending radius (BR). Figure 4 shows the strain data collected from the fibre aligned parallel to the CHS and from the fibre wound helically around the CHS. For these experiments, the optical fibre sensor intersected the mid-plane at 0° rotational angle.

Usually, BOTDR technology is used to monitor the engineering structures subjected to small deformations such as bridges, pipelines, tunnels, and dams. However, in this study, the CHS experienced large bending deformations, resulting in relatively high strains. As shown in Figure 4a, the three-point bending under Level 5 deformation showed a strain value of 9782 µε (0.98%) at the midpoint. Increasing the frequency range for the Brillouin acquisition enabled measuring higher strain values by BOTDR. The material property data of extrusion-grade polypropylene materials published by the MatWeb database indicates an average elongation at yield of 12.8% [27]. Therefore, all experiments conducted are considered to fall within the elastic limit of the material.

As a cylindrical member undergoes bending, at a certain cross section, the points along its circumference experience varying strain magnitudes. This variation can be effectively represented as a sinusoidal function of strain with respect to the azimuthal angle. Maximum tensile strain occurs on the convex side of the bend, while the concave side experiences maximum compressive strain. As shown in Figure 4a,c, the fibre parallel to the CHS, attached to the convex side, sensed varying tensile strain along the length of the CHS. As shown in Figure 4b,d, the fibre wound helically around the CHS experienced both tension and compression strains, depending on its position along the CHS. It was clear that the strain increased proportionally with the level of deformation. Local strain peaks near the supports may be attributed to variations in the local bending radius, the presence of shear forces, and cross-sectional warping. Additionally, boundary conditions near the ends can introduce stress concentrations and non-uniform bending moments, further contributing to any localized strain anomalies.

**Figure 5:**
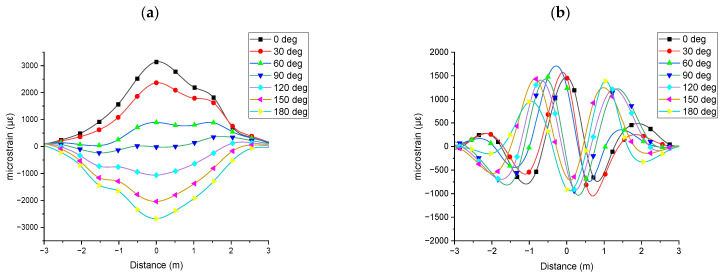
Experimental strain results obtained from the optical fibre sensors at varying rotational angles based on the mid-plane intersection point, measured during a three-point bending test under Level 1 deformation: (**a**) fibre parallel to the CHS axis; (**b**) fibre in helical winding around CHS.

For applications such as subsea power cable monitoring, understanding the direction of bending deformation is essential for identifying environmental changes and assessing their impact on the cable’s performance and lifetime. Figure 5 shows the strain results obtained from the optical fibre sensors at varying rotational angles based on the mid-plane intersection point. The top dead centre of the mid plane was considered as 0°. Clockwise rotational angles of 30°, 60°, 90°, 120°, 150°, and 180° were considered for the experimental and simulation studies. Both the fibre aligned parallel to the CHS and the fibre wound helically around it exhibited strain profiles characteristic of a three-point bending test under Level 1 deformation.

As shown in Figure 5a, when the strain was obtained by the fibre parallel to the CHS, the magnitude and the sign of the strain varied based on the respective rotational angle of the optical fibre. This represents the change in the direction of the bending deformation based on the location of the measured optical fibre. Figure 5b shows the strain obtained by the fibre wound helically around the CHS. In this case, the magnitude of the strain peak varied, and the strain profile shifted along the distance axis of the graphs (phase change), illustrating bending deformation relative to the optical fibre’s position.

## 4. Strain Response Simulation

Three-dimensional finite element analysis was performed using ANSYS 2024 R2 software. Static structural analysis was conducted to simulate the large bending deformations of CHS. The materials were assumed to be isotropic elastic. For the FEA, Young’s modulus and Poisson’s ratio of the material was 1.4 GPa and 0.4, respectively. The ANSYS static structural system was used with large deflection settings enabled for the analysis.

The CHS was analysed under three-point bending and constant curvature bending. Level 3 bending deformation was considered for both cases. For the FEA-based simulation, the length and the inner and outer diameters of the CHS were 6 m, 37.5 mm, and 40 mm, respectively. The mesh was constructed using quadratic mechanical elements with an element size of 5 mm. To simplify the simulation, fixed supports, forces, and moments were applied directly to the CHS without introducing any contact interactions. The intersection of the CHS’s outer surface and midplane was defined as a fixed support. As necessary, forces or moments were applied to the cylindrical edges on the outer surface of the CHS at locations along its axial length corresponding to points A and B shown in Figure 3. These edges encircle the CHS and intersect points A and B. In the three-point bending analysis, forces were applied perpendicular to the longitudinal axis of the CHS. To simulate constant curvature bending, moments were applied about an axis oriented in the radial direction of the CHS.

**Figure 6:**
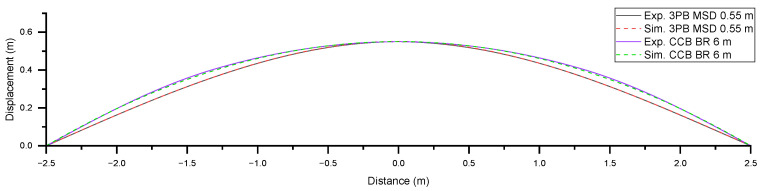
Displacement results obtained from experimental measurements and FEA-based simulation under Level 3 deformations of three-point bending and constant curvature bending.

Figure 6 shows the displacements (over the span ranging from −2.5 m to +2.5 m) of Level 3 bending deformations associated with experimental and simulation approaches. In the experimental study, the CHS was bent by adjusting the test rig parameters at certain points. Accordingly, the experimental displacements were obtained by using a measuring tool. In the simulation study, the CHS was deformed to a specific level by adjusting the applied forces and moments. Constant curvature bending was experimentally achieved using a seven-point bending setup. Although the induced curvature was not perfectly uniform, it closely approximated the ideal constant curvature profile. The results show that, at the corresponding deformation level, the simulation data correlated well with the experimental measurements for both the three-point and constant curvature bending scenarios.

**Figure 7:**
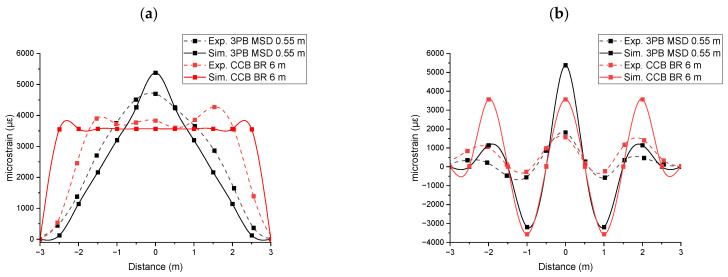
Strain results obtained from experimental measurements and FEA-based simulation for Level 3 deformation: (**a**) parallel to the CHS axis; (**b**) in helical winding around CHS.

Figure 7 presents the comparison of normal strain results obtained from experimental measurements and FEA-based simulation for Level 3 deformation. Strain measurements taken with the fibre aligned parallel to the CHS axis show that the three-point bending test produces a peak at the midpoint, while the constant curvature bending test results in a flatter strain curve. Strain measurements taken with the fibre wound helically around the CHS showed three tensile peaks at −2, 0, and 2 m, and two compression peaks at −1 and 1 m. This pattern occurred because the pitch of the helical winding was 2 m. When the CHS was subjected to three-point bending, the peak at 0 m was higher than the peaks at −2 and 2 m. In contrast, when the CHS was subjected to constant curvature bending, all peaks showed similar magnitudes. This distinction in strain profiles can be used to identify the shape of bending.

As presented in Figure 7a, the simulation and experimental data measured by the fibre aligned parallel to the CHS axis showed a close correlation. Under three-point bending, the simulation strain–distance response was relatively linear, compared to the experimental results. This discrepancy may be attributed to material nonlinearity, which was not accounted for in the FEA-based simulation. Under constant curvature bending, the experimentally measured strain profile between supports Q and S (−1.666 m to +1.666 m) exhibited a constant strain profile, as anticipated. However, the segments AQ and SB appeared relatively straight. This may be attributed to the unconstrained ends of the CHS during the experiment. In contrast, the simulation showed a constant strain along the entire curved length from point A to point B, which can be attributed to the applied moments at points A and B.

As presented in Figure 7b, the experimental strain measured by the fibre wound helically around the CHS was significantly lower than the point strain values obtained by the simulation. This discrepancy arises because the BOTDR system measures the Brillouin frequency shift as a spatially averaged value over the entire length of the optical pulse. Within the optical pulse which corresponds to a specific distance along the fibre (here corresponding to 0.5 m), the optical fibre experienced varying strain. The BOTDR system detects the backscattered signal integrated over the entire length illuminated by the optical pulse. Consequently, the measured Brillouin frequency shift represents an effective spatial average of the local frequency shifts within that spatial segment. When the strain is uniform over the optical pulse (respective fibre length), the measured Brillouin shift accurately reflects the local strain at that point. In contrast, if the strain varies within the segment, the result corresponds to an averaged value, potentially reducing the accuracy of the measurement.

**Figure 8:**
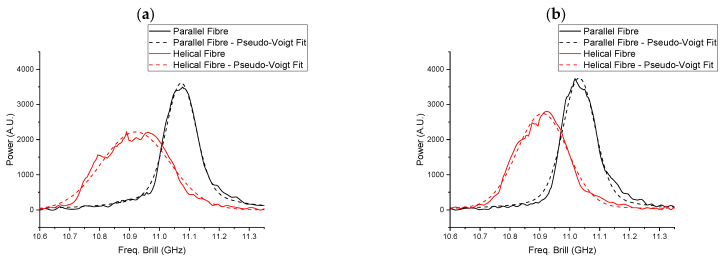
Brillouin spectrum corresponding to the measured strain at the CHS’s midpoint (0 m) under Level 3 deformation: (**a**) three-point bending; (**b**) constant curvature bending.

Figure 8 presents the Brillouin spectrum corresponding to the experimentally measured strain at the CHS’s midpoint (0 m) under Level 3 deformation. The data were fitted using a Pseudo-Voigt profile function to model the Brillouin gain spectrum. The Brillouin frequency shift and spectral width varied depending on the bending and layout configurations. As the spectral width increases, the shift of the peak position decreases, resulting in a reduced apparent strain variation. Table 2 presents the effect of CHS’s bending behaviour and fibre’s layout configuration on strain variation within the optical pulse, as well as their influence on the Brillouin spectral width. For analysis, the width of the Brillouin gain spectrum was evaluated at 10% of its maximum amplitude.

Each scenario was ranked based on the calculated Brillouin spectral width. As noted in Table 2, certain conditions lead to strain variation within the optical pulse, as indicated in the accompanying footnote (*). Both three-point bending behaviour and the helical fibre layout contribute to increased strain variation within the optical pulse. The CHS subjected to three-point bending, with data acquired using a helical fibre layout, exhibited the greatest error in point strain measurements (Rank 1). Figure 7b (3PB) also provides a clear illustration of the comparison between the experimental and simulation data, highlighting the scenario with the highest difference. In contrast, the CHS under constant-curvature bending, with measurements taken using a parallel fibre layout, demonstrated the highest accuracy in point strain comparison (Rank 4). Figure 7a (CCB) clearly illustrates the comparison between experimental and simulation data, emphasizing the scenario with the lowest difference. In a uniform strain field, the Brillouin gain spectrum is sharp and symmetric. In contrast, in a non-uniform strain field, the BGS becomes distorted, broader, and skewed.

The experimental data obtained from the helically wound fibre reflect the strain measured along the fibre’s helical path. Since the helical path is longer than the actual (axial) length of the CHS, this introduces a slight mismatch between the spatial references of the experimental and simulation data. In this case, the helical length for one complete turn is 2.004 m, while the axial (pitch) length is 2 m. This results in a mismatch of 4 mm per turn between the helical path and the axial distance. This discrepancy becomes more pronounced with increasing CHS diameter and a greater number of helical turns. Additionally, reducing the helical pitch could further increase the difference between the helical length and the actual axial length of the cylindrical member. Therefore, measuring the bending behaviour of cylindrical members using helically wound fibres requires corrections in both strain and distance measurements. Strain measurement errors arise due to the spatial average of the frequency shift effect along the helical path, while distance measurement errors result from the discrepancy between the helical length and the actual axial length.

**Figure 9:**
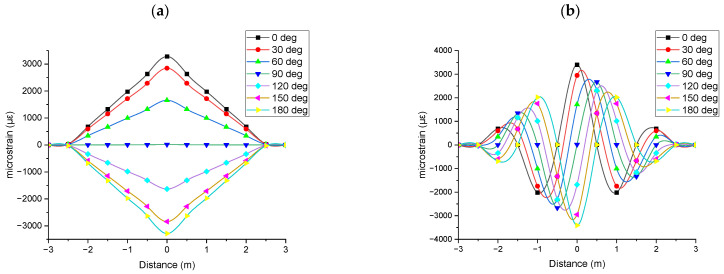
Simulation strain results at varying rotational angles based on the mid-plane intersection point, measured during a three-point bending test under Level 1 deformation: (**a**) parallel to the CHS axis; (**b**) in helical winding around CHS.

Finite element analysis was further extended to study the direction of the bending. Similar to the experimental study, a three-point bending analysis conducted under a Level 1 bending deformation. Normal strain was evaluated by considering the mid plane intersecting point of the optical fibre sensor. The top dead centre of the mid plane was considered as 0°. Clockwise rotational angles of 30°, 60°, 90°, 120°, 150°, and 180° were considered for the analysis. Figure 9 shows the strain results obtained from the simulation by considering the varying rotational angles based on the mid-plane intersection point. The simulation results shown in Figure 9 are more distinct strain profiles compared to the experimental results in Figure 5. Both experimental and simulation results showed similar strain profiles. Measurements from fibres aligned parallel to the axis exhibited changes in magnitude and sign, while those from helically wound fibres exhibited variations in both magnitude and phase.

## 5. Strain Response Inverse Analysis

The strain data acquired from the BOTDR has been used for an inverse analysis to reconstruct the deformed shape of the CHS. The following theoretical approach was employed for this analysis. It was assumed that the deformation of the CHS was only bending and occurs in one plane. The general geometrical relationship between the tilt angle of the CHS, θ, and the length along the CHS central axis, s, has the following form:

(2) $$\frac{d\theta }{ds}=\frac{1}{R}$$

where R is the curvature radius of the CHS. It was also assumed that the maximum curvature radius of the CHS is much less than its transverse radius. In this case, the strain at the external radius of CHS, where the optical fibre is attached is given by

(3) $$\varepsilon =\frac{a}{R}$$

where a is the CHS radius. By substituting this equation into (2), we obtain

(4) $$\frac{d\theta }{ds}=\frac{\varepsilon }{a}$$

Integrating this differential equation gives the curvature radius along the CHS:

(5) $$\theta (s)=\frac{1}{a}\int \varepsilon \;ds$$

Next, by changing from the coordinate system of the CHS to the Cartesian coordinate system:

(6) $$dx=\cos\theta \left(s\right)ds$$

(7) dy=sinθ(s)ds

Here, x and y are the horizontal and vertical coordinates, respectively. Integrating (6) and (7) gives (8) and (9), the parametric equations of the curve representing the shape of the CHS:

(8) $$x(s)=\int \cos\theta \left(s\right)ds$$

(9) y(s)=∫sinθ(s)ds

**Figure 10:**
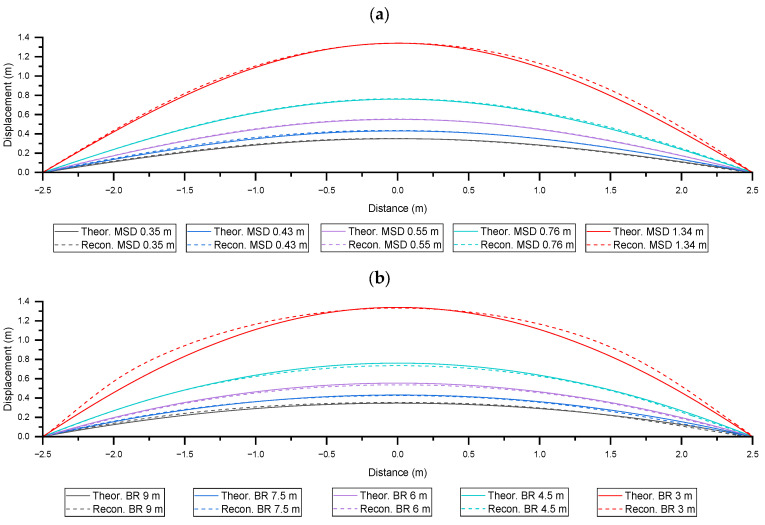
Reconstructed shapes of the CHS based on the experimental strain data obtained by the fibre parallel to the CHS: (**a**) three-point bending; (**b**) constant curvature bending.

Figure 10a,b show the reconstructed shapes of the CHS using the experimental strain data presented in Figure 4a,c, respectively, obtained by the fibre parallel to the CHS axis. For the reconstruction of shape of CHS, the constants defining the shape were adjusted. Accordingly, the span between the opposing fixed points was set to 5 m, and the maximum elevation of Level 5 bending was set to 1.34 m. The results showed acceptable agreement with the theoretical displacement profiles. In addition, the model of shape reconstruction that we used is nonlinear and is not limited to linear geometric deformations. Therefore, the shape reconstruction does not introduce any inaccuracies related to large deformations.

For the helical fibre, the strain is modulated along the CHS by a sinusoidal function. Since there are data for different angles of rotation, it is possible to find the strain along the CHS from modulated data with different phase shifts. Equations (10) and (11) consider two data sets obtained at two rotation angles with 90 degrees (orthogonal) between them:

(10) $$b_{1}\left(s\right)=\;\varepsilon \left(s\right)sin\left(\frac{s}{\Lambda }+\varphi \right)$$

(11) $$b_{2}\left(s\right)=\varepsilon \left(s\right)sin\left(\frac{s}{\Lambda }+\varphi +\frac{\pi }{2}\right)$$

Here, $\varepsilon \left(s\right)$ is the strain along the CHS, Λ is the helical pitch, and φ is the respective phase. Taking the root from the sum of squares of these two components, the absolute value of the demodulated strain can be obtained as given in (12), and it does not depend on the phase:

(12) $$\sqrt{{b_{1}\left(s\right)}^{2}+{b_{2}\left(s\right)}^{2}}=\left|\varepsilon \left(s\right)\right|$$

**Figure 11:**
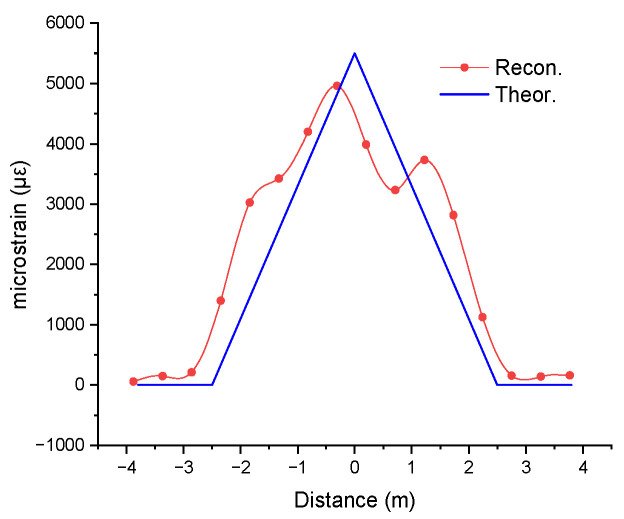
Demodulated strain obtained from the measurements with the helical fibre, compared to the theoretical curve.

**Figure 12:**
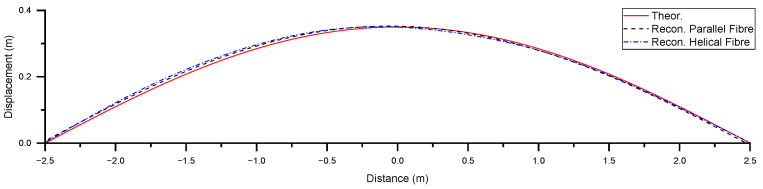
Displacement results for a Level 1 deformation under three-point bending, obtained from shape reconstruction using parallel and helically wound fibres, compared with the theoretical displacement.

Figure 11 shows the demodulated strain obtained from the measurements with the helical fibre. The averages of three curves for the pairs of angles 0 and 90°, 30° and 120°, and 90° and 180° were obtained. The curve for the demodulated strain corresponds well to the theoretical one. The demodulated strain from the helically wound fibre was used to reconstruct the shape of the CHS, following the same method used for the fibre aligned parallel to the CHS. Figure 12 shows the displacement results from shape reconstruction using parallel and helically wound fibres, compared with the theoretical displacement curve for a Level 1 deformation under three-point bending.

**Figure 13:**
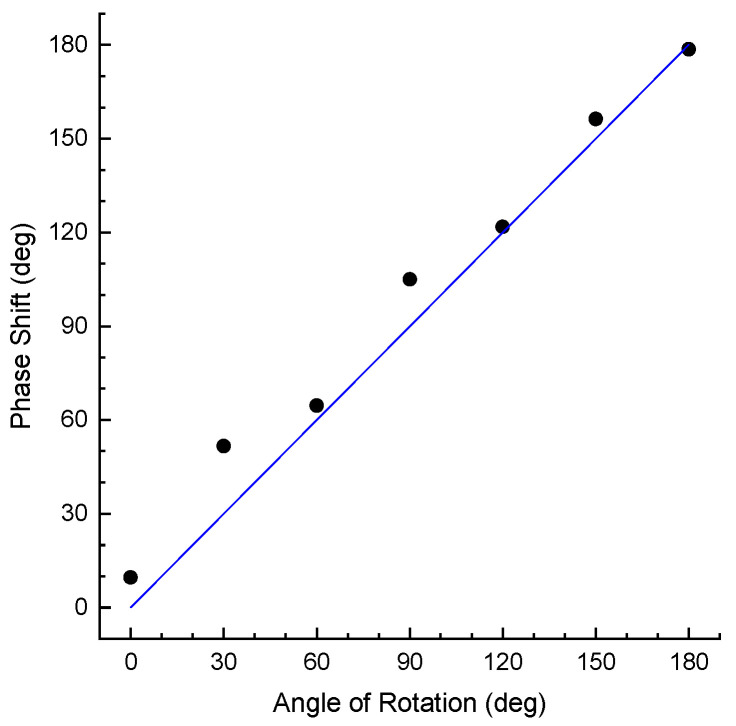
Phase shift of the strain curves for helical fibre as a function of rotation angle. The straight line is the ideal case.

The strain data measured from the helical fibre with different angles of rotation can also be used to reconstruct this angle. The angle of rotation determines the direction of bending and the phase of each curve in Figure 5b. The phase of the strain curve of the helical fibre can be analysed to find the angle of rotation. To achieve this, fast Fourier transform was applied to every curve in Figure 5b and the angle of the complex number at the spatial frequency corresponding to the helicity period of the fibre was obtained. The results are presented in Figure 13, which shows the phase shift of the curve as a function of rotation angle. The dependence appears to be nearly linear. The mean deviation from the correct angle is 8 deg. Thus, the strain data from the helical fibre can be effectively employed to measure the direction of CHS bending.

The minimum number of straight sensing fibres required for 2D reconstruction is one when the fibre is not located in the neutral plane of the bended structure. If the plane of the 2D bending is unknown prior to fibre installation, at least two straight fibres are needed in two orthogonal planes to determine the bending direction. This task of 2D bending in an unknown plane is more related to 3D reconstruction, which is not considered here. For helical fibres, one sensing fibre provides information about strain when it is going outside of the neutral plane of the bended structure. Thus, two quarters of helical pitch along the fibre provide useful information about the strain, while the other two quarters do not. Therefore, at least two helical fibres are needed to perform 2D reconstruction. On the other hand, the same two helical fibres would be sufficient for 3D reconstruction. Additional fibres provide more information about the strain distribution, offering better coverage of the structure length and increasing the accuracy of reconstruction.

## 6. Conclusions

This study evaluates the three-point bending and constant curvature bending of slender cylindrical structures using distributed strain sensing and finite element analysis. Strain measured with the fibre aligned parallel to the longitudinal axis and with the fibre helically wound around the CHS increased proportionally with the level of deformation. When the strain was obtained from the fibre aligned parallel to the CHS, the magnitude and sign of the strain varied based on the respective rotational angle of the optical fibre. When the strain was obtained from the fibre wound helically around the CHS, the magnitude of the strain peak varied, and the phase of the profile changed, illustrating the direction of the bending deformation relative to the optical fibre’s position. Both the parallelly aligned and helically wound fibres showed distinct strain profiles that differentiate the three-point bending and constant curvature bending behaviours. Strain measured by the parallelly aligned fibre showed a good correlation with the FEA. The experimental strain measured by the fibre wound helically around the CHS was significantly lower than the simulated values. This discrepancy arises because the BOTDR system measures the Brillouin frequency shift as a spatially averaged value over the entire length of the optical pulse. The impact of averaging in BOTDR scanning can be reduced by employing a low optical pulse (shorter averaging length) and using a high helical pitch when attaching the sensing fibre around a cylindrical structure.

BOTDR revealed precise, long-range sensing with absolute strain measurements, which are critical for shape reconstruction of elongated structures, surpassing alternative DOFS technologies. The strain data acquired from the BOTDR can be used for inverse analysis to reconstruct the deformed shape of the cylindrical structures. The strain data measured from the helical fibre with different angles of rotation can be used to determine the direction of bending. Overall, this study establishes a foundation for using BOTDRbased distributed strain sensing to monitor large deformations in long, slender cylindrical structures. It compares parallel and helical fibre layouts under three-point and constant curvature bending conditions, introduces a novel method for evaluating deformations, and offers practical guidance on fibre configuration while addressing key limitations, such as spatial averaging effects. The insights gained from this research contribute to the development of sensing systems for critical cylindrical infrastructure, such as subsea power cables.

## Figures and Tables

**Table 1:** Fixed and variable dimensions on the test rig for different levels of bending deformations.

Level of the Bending Deformation	Fixed Dimensions (m)		Variable Dimensions (m)				
			Three-Point Bending	Constant Curvature Bending			
	AB (Span)	AP, PE, EC, CG, GR and RB	CD (Midspan Displacement)	Bending Radius	CD	EF and GH	PQ and RS
Level 1	5	0.833	0.35	9	0.35	0.32	0.20
Level 2			0.43	7.5	0.43	0.38	0.24
Level 3			0.55	6	0.55	0.49	0.31
Level 4			0.76	4.5	0.76	0.68	0.44
Level 5			1.34	3	1.34	1.22	0.84

**Table 2:** Effect of bending behaviour and fibre layout on strain variation within the optical pulse.

Bending Behaviour	Fibre Layout	Width of the Pseudo-Voigt Fit at 10% Full Height	Rank (Highest Best)
Three-point bending *	Parallel	0.29	3
Three-point bending *	Helical *	0.49	1
Constant curvature bending	Parallel	0.27	4
Constant curvature bending	Helical *	0.38	2

* Induces strain variation within the optical pulse.

## Data Availability

Data supporting this study are available from the corresponding authors upon request.
